# Family-related risk indicators and dental attendance in association with dental caries in preschool children

**DOI:** 10.1186/s12903-024-04870-x

**Published:** 2024-09-14

**Authors:** Anna-Maria Pelkonen, Päivi Rajavaara, Hannu Vähänikkilä, Vuokko Anttonen, Marja-Liisa Laitala

**Affiliations:** 1 Research Unit of Population Health, University of Oulu, Oulu, Finland; 2 The Wellbeing Services County of North Ostrobothnia, Pohde, Finland; 3 Medical Research Center Oulu, Oulu University Hospital and University of Oulu, Oulu, Finland; 4 Arctic Biobank, Infrastructure for Population Studies, Faculty of Medicine, Northern Finland Birth Cohorts, University of Oulu, Oulu, Finland

**Keywords:** Dental caries, Risk indicators, Family-related factors, Family functioning, Dental attendance, Preschool-age, Children

## Abstract

**Background:**

Determining risk indicators behind dental caries is important for identifying children in need of enhanced dental care. The aim of this register-based study was to investigate the association of family-related risk indicators and dental attendance in the development of dental caries in preschool children.

**Methods:**

The data for this study were collected from the medical records of 206 randomly chosen preschool children who lived in the city of Oulu, Finland, during 2014–2020. Data on challenges in family life, missing appointments and utilization of oral health care were collected. Sum functions were formed of risk indicators. Analyses were carried out for three age groups (1- to 2-, 3- to 4- and 5- to 6-year-olds) born between 2014 and 2018.

**Results:**

There was a significant association between the number of family-related risk indicators and the prevalence of manifested caries lesions in the age group of 5- to 6-year-olds. All family-related risk indicators and information about health care utilization were associated with dental caries risk. Challenges in a child’s family life were present among 20.3% of the 5- to 6-year-olds. In all age groups, the most common challenges in family life were parental exhaustion and problems in the parents’ relationship or divorce.

**Conclusion:**

Family-related risk indicators and dental attendance should be considered when determining caries risk. The caries risk indicators investigated here are associated with each other.

## Introduction

According to WHO’s Global Action Plan on Oral Health by 2023 [1] strategic objectives, it is necessary to promote oral health and to prevent oral diseases, by addressing their risk factors. To understand and control dental caries as a disease, the underlying risk factors must be understood. Pitts et al. [2] presented a division of dental caries risk factors into two categories: patient and intraoral level risk factors. At patient/family level, these risk factors are, e.g., recently treated dental caries lesions in parents or siblings [3], DGA need in the family [4], and family functioning [5]. At intraoral level, the risk factors include teeth with caries lesions as well as its severe consequences such as extractions or pufa (pulpitis, ulceration, fistula, or abscess caused by dental caries) [6].

Family functioning has been found to play a significant role in children’s well-being. Family-related risk factors have been noted to expose children to a variety of health problems. Impaired family functioning has been associated with psychiatric disorders [7], overweight and obesity from early to mid-childhood [8], higher sugar consumption of preschool-aged children [9] and poor eating habits [10]. Moreover, Duijster et al. [11] suggested that family functioning may be a mediator of socio-economic inequalities in children’s oral health. Long-term need for family social assistance has also been found to increase the risk of dental caries and missing appointments at dental health care [12].

Parenting practices and family interaction have also been shown to be associated with dental caries [13]. Duijster et al. [5] showed that children in families without problems in functioning had on average lower dmft-scores compared to children living in more poorly functioning families. This may be explained by oral hygiene behaviors. The same trend was seen in a study of Almutairi et al. [14] where unhealthy behavior control was associated with greater number of dental caries lesions diminishing a child’s oral health-related quality of life (OHRQoL). However, a study by Bilal et al. [15] did not find a direct association concerning family functioning and OHRQoL in association with dental caries.

Adolescents’ dental avoidance has been associated with higher dental caries experience (DMFT) [16] and more invasive dental treatment [17]. A Norwegian study [17] noted that sociodemographic load and dental fear or behavior management problems may lead to avoidance of dental care. In addition, caries risk indicators were associated with past oral health problems and missed appointments [18].

In Finland, public health care including oral health care for all children under 18 years, is equal and free of charge in all parts of the country. According to the outpatient notification register of primary health care (Avohilmo), 99.5% of families with children under school age use public child health clinic services [19]. At least three statutory oral health examinations are offered for children under school age (1- to 2-year-olds, 3- to 4-year-olds and 5- to 6-years-olds) and additionally, according to individual need [20]. Generally, dental caries experience is low among Finnish children. However, it has been suggested that caries is still common among certain subpopulations [21] that should be identified and targeted for preventive dental care. So far, the research on the association of family-related indicators with dental caries in preschool children is scarce.

The aim was to study the association of family-related risk indicators and dental attendance in association with dental caries prevalence and experience in preschool children considering also the most severe consequences of dental caries. Challenges in family functioning, missing appointments and utilization of oral health care were also targets of interest.

## Materials and methods

### Data collection

This data-based study was designed and written according to the STROBE Checklist [22]. The material was collected from the medical records of 206 randomly chosen children under 7 years who had lived in the city of Oulu, Finland, during the entire study period during the years 2014–2020. Data on all three statutory clinical oral examinations were collected of children at the age of 5–6 years (children born in 2014), on two examinations at the age of 3–4-years (children born in 2014 and 2016) and one examination at the age of 1–2 -years (children born in 2014, 2016 and 2018). The total numbers of children in those age groups living in Oulu were 2456, 2249 and 2023, respectively. This study design allowed monitoring caries development longitudinally in the oldest age group (born in 2014). None of the children were excluded due to potential problems with general health, chronic diseases or physical or mental disabilities.

Data collection was performed from March 2022 to July 2022. The randomization of the study population was performed using a number generator (Calculator.net). The sample size of the study was confirmed by power calculations assuming that the difference in decay value between the groups d = 1.2─0.7 = 0.5. With the significance level alpha = 0.95 and power 1-Beta = 0.8, the sample size was confirmed to be 54 per age group. Data collection was continued until at least 54 children from each age group with statutory examinations were obtained. Those children who did not have statutory examinations were not excluded from the study, thus, the final number of children in the study was 206.

The background information included the child’s year of birth and gender (*f/m/othe*r). The information collected from the medical and dental records comprised children’s visits in dental care (*number of visits*, both *clinical oral examinations and treatments*), the profession of the person performing the examination/treatment (*dentist/oral hygienist/dental nurse*), and clinical findings (*number of teeth with initial or dentinal caries lesions* and *dmf indices*). The presence of the recorded caries risk indicators was registered (*yes/no*) according to earlier validation and Caries (control) Current Care Guidelines in Finland [23]. The risk indicators related to previous caries history comprised *pufa index (number of teeth with signs of pulpitis*,* ulcerations*,* fistula and abscesses; oral conditions resulting from untreated caries) and number of extracted teeth due to caries* as well as DGAs (dental general anesthesia) treatments (*yes/no*).

The information on challenges in family life, cancelled appointments (*n*) and no shows (*n*) in public child health clinic of all included children was collected from the records of public health nurses (*n* = 206) as well as oral health care, including those who did not have statutory clinical dental examinations. The data of possible challenges in family life were recorded as follows: *parental exhaustion*,* sleeping or behavioral problems of a child*,* death or grief in the family*,* need for support from social services or child living in out-of-home care*,* guardian’s unemployment or self-reported problems with mental health*,* divorce of parents or problems in the parents’ relationship*. Challenges were recorded (*yes/no*) every time when mentioned.

### Statistics

Cumulative, descriptive information of the participants at the age 1–2, 3–4 and 5–6 years in the background information and oral health and dental attendance was given. The results of the analyses were described as frequencies and distributions by mean values (standard deviation, SD), proportions and graphically. Associations between the initial and dentinal caries lesions (i, d) and other variables were analyzed using the Chi-square or Fisher’s exact test and t-test. A statistically significant difference between the groups was described by *p* < 0.05. The statistical analyzes were performed using IBM SPSS Statistics for Windows, Version 29.0 (Armonk, NY: IBM Corp).

### Ethical considerations

Permission for the study was obtained from the register holder, the City of Oulu (Nov 19, 2021, registration authorization number OUKA/12798/07.01.04.02/2021). According to Finnish legislation (Data Protection Act 1050/2018), a statement from the regional medical research ethics committee of the Wellbeing Services County of North Ostrobothnia was not required. The data were stored and analyzed protecting the anonymity of the participants by creating ID numbers, the key for which was held by the first author.

## Results

Challenges in a child’s family life were present among as many as 20.3% of the five-year-olds (Table 1) and 73.7% of the children in this age group had challenges in their family life recorded at least twice during the follow-up period. The most common reasons for challenges in family life were parental exhaustion and problems in the parents’ relationship or divorce (Table 1). Missed appointments were found in 19.9% of the participants in child welfare care and 16.8% in dental care. Dental general anesthesia (DGA) was performed on average for 1.5% of the study population (Table 2). The risk indicators associated with at least initial caries lesions are presented in Table 3.

**Table 1:** Challenges in family life according to different age groups

Challenges in family life at different age (n)			
	1–2 years n = 206	3–4 years n = 142	5–6-years^a^ n = 69
Self-reported mental health problems of a parent	1	1	1
Sleeping or behavioral problems of a child	2	4	2
Parental exhaustion	10	5	2
Family having support from social services	4	4	3
Problems in relationship of parents or divorce	10	8	5
Other burdening factor of the family (e.g., sorrow, unemployment)	3	3	0
Out-of-home care	1	2	1

^**a**^ 73.7% children of this age group had challenges in their family life recorded at least twice during the follow-up period

There was no statistically significant difference in dental caries manifestations between the genders (Table 4). In the oldest age group, skewness in dmf distribution was seen if d < 2. Prevalence of teeth with dentinal and initial caries lesions > 1 was significantly (*p* < 0.001) associated with previous caries history, pufa index > 0 and prevalence of extracted teeth due to dental caries (> 0) in all age groups.

**Table 2:** Distribution of the participants according to gender and non-clinical variables studied

Variable	Proportions and numbers of the study population at different age (%, (*n*))		
	1–2 years n = 206	3–4 years n = 142	5–6 years n = 69
Gender male female	49.4 (102) 50.6 (104)	45.8 (65) 54.2 (77)	47.6 (33) 52.4 (36)
At least one challenge in a child’s family life	15.0 (31)	19.0 (27)	20.3 (14)
No statutory examination	21.4 (44)	18.3 (26)	8.7(6)
At least one treatment under dental general anesthesia (DGA)	1.0 (2)	2.1 (3)	1.4 (1)
At least one missed appointment in dental care	21.4 (44)	19.0 (27)	10.1 (7)
At least one missed appointment in public child health clinic	21.8 (45)	19.0 (27)	18.8 (13)

**Table 3:** Caries-related clinical findings at different age

Clinical findings	Descriptives of clinical findings at different age		
	1–2-years n = 164	3–4-years n = 118	5–6-years n = 63
i, d ^**a**^ (%,(n))	4.3 (7)	15.3 (18)	30.2 (19)
> 0 extracted tooth due to dental caries (%, (n))	1.2 (2)	2.5 (3)	7.9 (5)
pufa > 0 (%, (n))	1.8 (3)	3.4 (4)	11.1 (7)
dmf (mean (range))	0.02 (0, 2.00)	0.2 (0, 6.00)	0.8 (0, 13.00)

^**a**^ initial and dentinal caries lesions

**Table 4:** Association of initial and dentinal caries lesions (i, d) in primary teeth with challenges in family life as well as dental attendance related variables

Variable	Number of children at different age (*n*)								
	1–2 years n = 164			3–4 years n = 118			5–6 years n = 63		
	i, d = 0	i, d > 0	p*	i, d = 0	i, d > 0	p*	i, d = 0	i, d > 0	p*
Challenges in family life ^a, b^	22	0	> 0.999	12	7	0.010	7	5	0.485
Dental general anesthesia (DGA)	0	2	< 0.001	0	3	< 0.001	0	1	0.302
Missing appointments in child welfare clinic	29	4	0.031	16	4	0.734	5	7	0.033
Missing appointments in dental care ^b^	1	1	0.024	3	1	0.011	0	1	0.302
Symptom- driven dental attendance	14	3	0.025	12	4	0.265	6	3	> 0.999

* Fisher Exact Test

^**a**^ Data were collected from all children (1–2 years n = 206, 3–4 years n = 142 and 5–6 years n = 69)

^**b**^At the age of 1–2-years, 44 children did not have clinical examinations or diagnosis of dental caries. At the age of 3–4 years, 26 children, and at the age of 5–6 years, 6 children did not have a clinical examination or diagnosis of dental caries

**Fig. 1:**
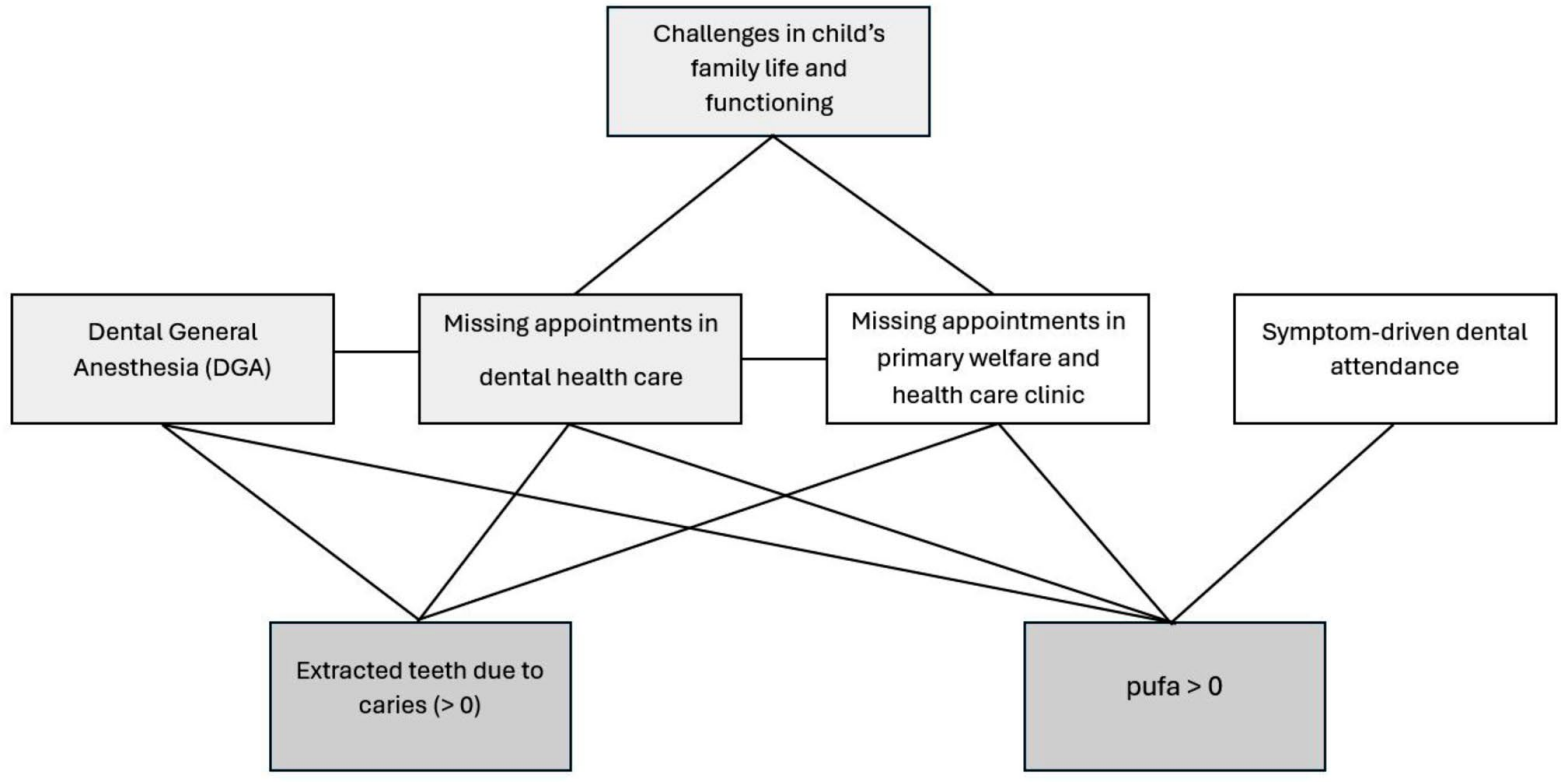
Statistically significant associations (lines between the boxes indicating significant differences, *p* < 0.05) between challenges in a child’s family life and dental attendance as well as severe consequences of dental caries (pufa > 0 (number of teeth with signs of pulpitis, ulcerations, fistula and abscesses) and teeth extracted (> 0) due to dental caries)

**Fig. 2:**
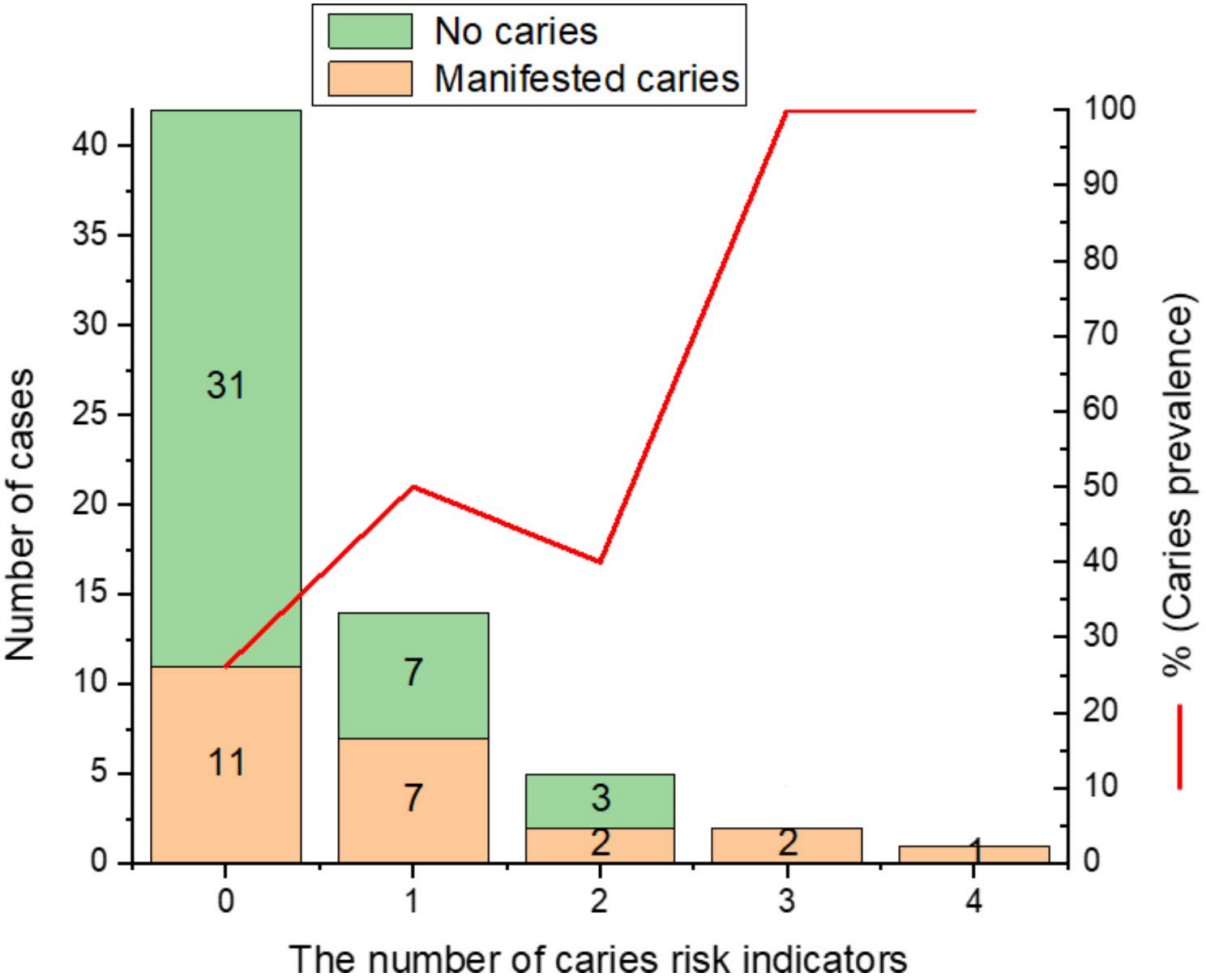
The number of family-related risk indicators in 5- to 6-year-olds in association with the proportion of children with dental caries prevalence (initial and dentinal caries lesions (i, d) > 0)

In the age group of 1–2 years, missing appointments at dental health care clinic was associated (*p* < 0.05) with DGA, pufa index > 0, prevalence of caries lesions and extracted teeth due to dental caries (Table 3; Fig. 1). At that age, children with dentinal caries lesions had more symptom-driven dental attendance and pufa index > 0 than those with sound teeth (*p* < 0.05). Missing appointments were also associated with prevalence of caries lesions, pufa index > 0 and DGA in the age group of 3–4 years (*p* < 0.05) (Table 3; Fig. 1). At the age of 3–4 years, children who had symptomatic-driven dental attendance had more often pufa index > 0 (*p* < 0.05). (Table 3; Fig. 1). In the age group of 5–6 years, 52.2% of the children with initial or dentinal caries lesions had a history of missing dental health care appointments. In the age group of 5-6-years, there was a significant association between the number of caries risk indicators and the prevalence of initial or dentinal caries lesions (*p* < 0.001). All children in the age group of 5–6 years having at least three risk indicators (*n* = 3) had manifested caries lesions (Fig. 2).

Challenges in family life were associated with severe consequences of dental caries. Statistically significant associations (*p* < 0.05) between variables studied are presented in Fig. 1 showing the multifactorial character of dental caries.

## Discussion

There is still need for new aspects and findings in the fight against one of the world’s most widespread, polarizing, and economically burdening diseases that impairs the quality of life of children and their families [24]. Here, as many as around 20% of the children had challenges in family life; in some families, challenges persisted for years. All the analyzed family-related risk indicators and information about dental attendance (challenges in family life, missing appointments at child welfare clinic or dental health care clinic and symptom-driven dental care attendance) were found to be associated with dental caries. In addition, previous caries history measured as severe consequences of dental caries (pufa index > 0 and the number of extracted teeth due to dental caries and DGAs) was found to associate significantly with caries prevalence. Our finding is in line with earlier studies [4, 6, 25]. Of course, pufa, extractions and DGA can be considered as consequences of untreated caries, but they can also tell about patient´s future caries risk. This study highlighted the connections between different risk indicators. All these risk indicators were clearly connected to each other, which may have a cumulative effect on dental caries risk, as well as on other threats to the child’s well-being.

Along with the traditional ones, risk indicators related to challenges of family life, family functioning [5, 8, 9, 11, 13, 14, 26] and general life management [4, 11] have emerged, manifesting, for example, as the need for DGA and missing health care appointments. Dental attendance and the family-related risk indicators in question are also in many ways linked to dental caries [5, 13, 14]. This was shown here and in the study of Wigen et al. [18] in 5-year-old children. It is especially important for oral health care professionals to be aware of this association. So far, this perspective has received less attention when studying the causes of dental caries in children. However, it should be kept in mind that also dental fear can be a reason for missing appointments [27] and genetics (epigenetics) can play a role in caries risk [28, 29].

Families having challenges in family life are vulnerable in many ways. Stressful periods and situations in life threaten children’s wellbeing [30]. Challenges in family life can mean that parental skills or resources are not in balance with children’s needs. This loading situation can lead to loss of control over life and among other things, to neglect of adequate oral hygiene, eating habits and missed dental appointments thus threatening the overall well-being of families. In this study, we were not able to measure family functioning in a validated way, but the challenges in family life recorded by health and dental health professionals and dropping out of health care services gave clear indications of it. In Finland, maternity and child health clinic employees must determine whether the family’s resources are sufficient [20] but assessing this requires the presence of a professional with appropriate skills and training. In this study, according to child’s age, some challenges in family life were recorded in as many as one fifth of the families which means that this burden is very common. Literature on this topic is scarce.

One of the main findings of this study was the clear relationship between the number of risk indicators and the prevalence of manifested caries lesions. This connection seems to be obvious, but despite this, the importance of determining family-related and dental attendance related risk indicators is still undervalued. It is important for oral health care professionals to record potential risk factors in patient records and consider the risk in determining, e.g., the length of the recall interval and a plan for preventive care.

In Finland, data related to oral health are collected comprehensively and monitoring data are available for research. One of the strengths of the study is that Finland has an oral health care service system covering the entire population. For the different age groups included in the study, information was obtained about the oral health status of children of different ages, although some of the study groups remained rather small and conclusions should be drawn with caution. In addition to the cross-sectional study design, this study allowed monitoring caries development longitudinally in the oldest age group. Due to the comprehensive data set and randomisation, the study population can be considered a representative sample of children under school age in Northern Finland. Recordings on the family-related risk indicators are important but unfortunately, these were found to be more incomplete in dental registers than in the registers of public health nurses. This can be considered as a limitation of the study. A larger research population would enable more advanced analyses to be carried out from the perspective of polarization of dental caries, for example.

Based on the findings of this study, it would be important to assess the situation of all families in more detail when determining the risk for dental caries. Oral health care professionals could assume a more significant role in supporting the well-being of families and in providing more child-centered dental care.

## Data Availability

The datasets used and/or analyzed during the current study are available from the corresponding author on reasonable request.
